# Assembly and Curation of Lists of Per- and Polyfluoroalkyl Substances (PFAS) to Support Environmental Science Research

**DOI:** 10.3389/fenvs.2022.850019

**Published:** 2022-04-05

**Authors:** Antony J. Williams, Linda G. T. Gaines, Christopher M. Grulke, Charles N. Lowe, Gabriel F. B. Sinclair, Vicente Samano, Inthirany Thillainadarajah, Bryan Meyer, Grace Patlewicz, Ann M. Richard

**Affiliations:** 1 Office of Research & Development, Center for Computational Toxicology & Exposure, U.S. Environmental Protection Agency, Durham, NC, United States; 2 Office of Land and Emergency Management, US Environmental Protection Agency, Washington, DC, United States; 3 ORAU Student Services Contractor to U.S. Environmental Protection Agency, Office of Research and Development, Center for Computational Toxicology & Exposure, Oak Ridge, NC, United States; 4 Senior Environmental Employment Program, US Environmental Protection Agency, Research Triangle Park, NC, United States

**Keywords:** cheminformatics, pfas (perfluorinated alkylated substances), environmental chemistry, computational toxicology, web-based information

## Abstract

Per- and polyfluoroalkyl substances (PFAS) are a class of man-made chemicals of global concern for many health and regulatory agencies due to their widespread use and persistence in the environment (in soil, air, and water), bioaccumulation, and toxicity. This concern has catalyzed a need to aggregate data to support research efforts that can, in turn, inform regulatory and statutory actions. An ongoing challenge regarding PFAS has been the shifting definition of what qualifies a substance to be a member of the PFAS class. There is no single definition for a PFAS, but various attempts have been made to utilize substructural definitions that either encompass broad working scopes or satisfy narrower regulatory guidelines. Depending on the size and specificity of PFAS substructural filters applied to the U.S. Environmental Protection Agency (EPA) DSSTox database, currently exceeding 900,000 unique substances, PFAS substructure-defined space can span hundreds to tens of thousands of compounds. This manuscript reports on the curation of PFAS chemicals and assembly of lists that have been made publicly available to the community via the EPA’s CompTox Chemicals Dashboard. Creation of these PFAS lists required the harvesting of data from EPA and online databases, peer-reviewed publications, and regulatory documents. These data have been extracted and manually curated, annotated with structures, and made available to the community in the form of lists defined by structure filters, as well as lists comprising non-structurable PFAS, such as polymers and complex mixtures. These lists, along with their associated linkages to predicted and measured data, are fueling PFAS research efforts within the EPA and are serving as a valuable resource to the international scientific community.

## INTRODUCTION

### Background

Per- and polyfluoroalkyl substances (PFAS) are a large class of synthetic chemicals that includes the following well-known representatives: perfluorooctanoic acid (PFOA), perfluorooctanesulfonic acid (PFOS), and ammonium perfluoro-2-methyl-3-oxahexanoate (the chemical often referred to as GenX)^1^. Since the 1940s, PFAS have been manufactured and used in a wide variety of industries both in the United States and globally. PFAS are found in everyday consumer products such as food packaging, non-stick, stain repellent, and waterproof products, including clothes and carpets, as well as cleaning products and paints (Gluge et al., 2020). Thousands of distinct PFAS exist in commerce or have been detected in environmental samples. PFAS are also widely used in industrial applications and for firefighting, the latter in the form of aqueous film-forming foams (AFFFs) that are a major contributor to environmental contamination. Whereas PFOA and PFOS have been well characterized in terms of their hazard, little to no toxicity information exists for the vast majority of PFAS. Evaluating thousands of PFAS using traditional toxicity approaches, in turn, would be impractical, costly and time-prohibitive, as well as requiring extensive use of animals. Accordingly, the U.S Environmental Protection Agency (EPA) initiated a research program in 2018 to a develop risk-based approach for conducting PFAS toxicity testing to facilitate PFAS human health assessments. Concurrently, in 2019, the EPA published its Action Plan for PFAS, which outlined a multiprogram national research plan to address the challenges associated with this class of chemicals (“EPA’s Per- and Polyfluoroalkyl Substances (PFAS) Action Plan”; PFAS_Roadmap 2021) and advocated for the use of computational toxicology approaches to fill information gaps. EPA’s Action Plan for PFAS has since been superseded by publication of the PFAS Strategic Roadmap (and associated National Testing Strategy) (October 2021), which articulates a testing plan and commitments for the EPA to achieve during 2021–2024 (PFAS_Roadmap 2021). These initiatives all rely on the foundation of relevant PFAS lists to fuel and define the scope of data gathering, categorization, and modeling efforts.

A long-standing challenge to the PFAS community has been the lack of a consensus definition of what constitutes a PFAS. The basic structure of a PFAS consists of a carbon chain with substituted fluorine atoms replacing hydrogen atoms on the chain, and with different categories of PFAS chemicals possessing different substituents and functional groups within (e.g., ethers) or terminal to the chain. In one of the earliest attempts to apply structure-based boundaries to the term, Buck et al. (Buck et al., 2011) defined PFAS as aliphatic substances that “contain one or more carbon atoms on which all of the hydrogen substituents (present in the nonfluorinated analogues from which they are notionally derived) have been replaced by fluorine atoms, in such a manner that they contain the perfluoroalkyl moiety (-C_n_F_2n+1_^−^).” Of note, the moiety described implies a fully fluorinated terminal carbon, whereas the text definition does not explicitly indicate a terminal carbon. In 2018, the Organization for Economic Cooperation and Development (OECD 2021) published a Global Database of Per- and Polyfluorinated Substances that focused on chemicals containing a perfluoroalkyl moiety with three or more carbons (i.e., –CnF2n–, *n* ≥ 3) or a perfluoroalkylether moiety with two or more carbons (i.e., –CnF2nOCmF2m−, n and *m* ≥ 1) (OECD-PFAS 2018). OECD mentions the distinction of whether a terminal fully fluorinated carbon is needed by noting that in this study, the definition of a perfluoroalkyl moiety has been expanded from “(CnF2n+1–)” in (Buck et al., 2011) to “−CnF2n−” to include PFAS with both ends of the perfluoroalkyl moiety connected to a functional group. More generally, in the non-scientific media and literature, PFAS have either been described as fully fluorinated, or loosely described as “highly fluorinated,” but a definition of what constitutes highly fluorinated is generally lacking. Among other problems, such arbitrary conventions for defining PFAS have resulted in ambiguous terminology that creates barriers to clear and effective communication and thwarts the comparison and reproduction of studies.

Starting in 2015, with increased focus on the environmental and health concerns surrounding PFAS, EPA researchers within the National Center for Computational Toxicology (incorporated into EPA’s Center for Computational Toxicology (CCTE) in 2019) undertook a major effort to curate and structure-annotate several public lists in EPA’s DSSTox database (Grulke et al., 2019). The lists, gathered from within and outside of EPA, included the OECD Global PFAS Database and encompassed PFAS of potential concern based on environmental occurrence (through literature reports and analytical detection) and manufacturing process data, as well as lists of PFAS chemicals procured and queued for testing within EPA’s intramural research programs (Patlewicz et al., 2019). These lists were made publicly available on EPA’s CompTox Chemicals Dashboard (hereafter, the Dashboard) (Williams et al., 2017).

In 2018, to begin to assess the combined coverage of the Dashboard PFAS lists, the lists were merged to create the first version of EPA’s PFASMASTER list. This initial consolidated list contained over 5000 unique PFAS substances, with the majority associated with a Chemical Abstracts Services Registry Number (CASRN) and almost 4000 represented with a defined chemical structure, the remainder consisting of polymers, mixtures, and ill-defined substances. Hence, by virtue of its component list contents, the PFASMASTER list served to define a practical, bounded PFAS chemical space representing the interests of researchers and regulators worldwide. Despite containing significant structured contents, however, the initially constructed PFASMASTER list was ad hoc and not bounded by a clear PFAS structure definition. Subsequent efforts, to be described below, have used structure-based queries across the entire public DSSTox database to create versions of a PFASSTRUCT list whose contents span a clearly defined, structurally bounded space within DSSTox that is intended to serve a broad range of EPA programmatic needs.

In June 2021, EPA’s Office of Pollution Prevention and Toxics (OPPT) narrowed the definition of PFAS for proposed reporting and recordkeeping requirements under the Toxic Substances Control Act (TSCA). For that specific proposed rule, PFAS were defined as any chemical substance or mixture that structurally contains the unit R-(CF2)-C(F)(R′)R″. Both the CF2 and CF moieties are saturated carbons but none of the R groups (R, R′ or R″) can be hydrogen (TSCA Substances, 2020) Hereafter, this is referred to as the TSCA 2021 definition. That definition was also adopted by the EPA for the draft Drinking Water Contaminant Candidate List 5 (Drinking Water Contaminant Candidate List 5-Draft 2021).

In July 2021, OECD proposed a revised definition of PFAS to comprehensively encompass the known Universe of PFAS. The rationale was to create a general PFAS definition that would be coherent and consistent across compounds from a chemical structure perspective and would be easily implementable to distinguish PFAS from non-PFAS. They defined PFAS as “fluorinated substances that contain at least one fully fluorinated methyl or methylene carbon atom (without any H/Cl/Br/I atom attached to it), i.e., with a few noted exceptions, any chemical with at least a perfluorinated methyl group (–CF3) or a perfluorinated methylene group (–CF2–) is a PFAS.” This revised definition removes the requirement that the structure is entirely aliphatic, and only requires that the minimal fully fluorinated methyl or methylene group are saturated and aliphatic (OECD-PFAS 2018; OECD-PFAS 2021). The United States (U.S.) Congress used a similar definition in the National Defense Authorization Act for Fiscal Year 2020, defining PFAS as “man-made chemicals with at least one fully fluorinated carbon atom” (National Defense Authorization 2020). It should be obvious that different regulatory programs are using different definitions for what constitutes a PFAS chemical.

At the time of this writing, the Dashboard provides access to data associated with over 900,000 chemicals. These data can include *in vivo* and *in vitro* toxicity data, experimental and predicted properties, exposure data and an array of search capabilities to investigate the data. The assembly of the data has occurred over almost 2 decades and was initiated with the development of the DSSTox database (Grulke et al., 2019). The DSSTox database, under constant curation and expansion, is the underpinning for the Dashboard and serves as the primary integrator of chemistry-associated data and lists surfaced via the Dashboard (Dashboard_Lists 2021). Lists, in turn, are segregated according to specific categories (e.g., pesticides, hydraulic fracturing), or are associated with regulatory programs (e.g., TSCA inventory) or projects within EPA’s CCTE, such as the ToxCast high-throughput screening program (Kavlock et al., 2012). The ability to provide access to chemical lists via the Dashboard serves as an effective means to organize, communicate, and distribute data to the community. Building on this capability, we have devoted significant effort to the curation and structure annotation of PFAS chemical lists over the past several years. At the time of writing there are over 30 PFAS lists available for viewing and download on the Dashboard, ranging in size and scope from 8 PFAS chemicals detected in fluorinated HDPE (high-density polyethylene) containers (List_Pesticide_Packaging 2021) to lists containing thousands of chemicals based on substructural definitions and searches (PFASSTRUCT_Navigation 2021). The largest of these lists contains almost 11,000 chemicals. The number of PFAS introduced into commerce, or detected in the environment or biota, as well as data associated with these PFAS, has continued to expand over the years.

At the same time, various proposed working definitions of PFAS have made it challenging to produce a single definitive reference list of chemicals that could be shared with the community via the Dashboard and satisfy the varied needs of the research and regulatory communities. This manuscript provides an overview of the various approaches that have been taken in recent years to deliver a wide range of PFAS lists via the Dashboard, as well as an analysis of the types of chemicals that are included in the most recent iteration of the overarching PFASSTRUCT and PFASMASTER lists.

## METHODS

### External Per- and Polyfluoroalkyl Substances Lists

Registering an external PFAS list into the DSSTox database involves initial auto-mapping of source substance identifiers (typically CASRN and names) to existing DSSTox content, indicating the best DSSTox matches, and flagging possible identifier conflicts and missing content. The importance of both the need for, and approaches to performing systematic chemical structure curation have been discussed previously (Fourches et al., 2010), specifically in terms of developing curated datasets for the purpose of QSAR modeling. In the case of this work, the curation approaches proven over a period of almost 2 decades, and described in great detail in a previous publication (Grulke et al., 2019), were applied to the development of the lists described herein. Specific details include enforcing a strict 1:1:1 mapping of CASRN to a unique name and structure and the details of approaches for resolving conflicts; interested parties are pointed to our previous work to understand the curation approach in more details.

In the case of the OECD Global PFAS Database, for instance, chemical names and CASRN were initially mapped to existing DSSTox content, but the major portion of list substances had to be newly registered. This was also the case for several early, publicly sourced PFAS lists imported into DSSTox which were missing from the database. Newly registered PFAS substances were subject to expert manual curation review to add chemical structures and to ensure that CASRN and names were uniquely assigned and consistent with the assigned structure. By way of DSSTox registration and Dashboard public distribution, thousands of PFAS substances with chemical structures have enriched public domain databases, such as PubChem (PubChem 2021)and ChemSpider (ChemSpider 2021). In addition, PFAS presented some unique challenges for DSSTox curators. The majority of source chemical names from public PFAS lists were lengthy systematic names that in some cases exceeded 256 characters in length, which can lead to truncation errors when transferred among commonly used applications. During review, DSSTox curators manually converted thousands of these systematic names to “perfluoro-type” names, which are more human-readable and intuitive. An example is the OECD-listed substance with CASRN 52956-82-8 (DTXSID10880456), originally named “2-Propenoic acid, 3,3,4,4,5,5,6,6,7,7,8,8,9,9,10,10,11,11,12,12,13,14,14,14-tetracosafluoro-13-(trifluoromethyl)tetradecyl ester,” whose name was reduced to the DSSTox Preferred Name “2-(Perfluoro-11-methyldodecyl)ethyl propenoate.” [Note, these names can be confirmed to be equivalent by using the free OPSIN name-to-structure conversion application (OPSIN 2021)]. In total, more than 3100 PFAS substance names in the latest PFASSTRUCT file have been manually condensed in this manner to perfluoro-type names.

In part due to prevalence of long systematic names in public PFAS listings, DSSTox curators have also encountered a plethora of PFAS acronyms circulating in PFAS listings in the public domain. The most familiar of these are PFOA and PFOS, but even those are commonly applied not just to the parent neutral acid, but to the anion and various salts. DSSTox curators register such acronyms as synonyms, but label these short, domain-specific PFAS acronyms as “ambiguous” due to their inconsistent and unregulated application (see, e.g., PFPA, which is used to refer to two distinct compounds: Perfluoropropanoic acid and Perfluoropentanoic acid). Hence, in the Dashboard, PFAS acronyms are often linked to multiple substance records, which alerts the community to their non-unique nature.

### Dashboard Per- and Polyfluoroalkyl Substances Structures Lists

Based on a review of chemicals contained within DSSTox in March 2018, the first PFASSTRUCT list released was assembled using a set of substructure filter conditions designed to broadly identify PFAS chemicals. The filter conditions did not precisely match the definitions from Buck et al. or from the OECD, but were designed to be simple, reproducible, and transparent, yet general enough to encompass the largest set of structures having sufficient levels of fluorination to potentially impart PFAS-type properties. For this list (PFASSTRUCTV1 2021), the defined filters were: 1) formula must contain 4-1000 fluorine atoms; 2) structure must contain two adjacent CF2 groups, either in a chain or in a ring system; 3) fluorine-to-carbon ratio (#F/#C) must be ≥0.5; and 4) removal of Markush structures, charged species (e.g., anions), free radicals, and deuterium- and C13-labeled chemicals. Applying this set of filters across the entire DSSTox database, which at that time exceeded 700,000 chemicals, led to an initial PFASSTRUCTv1 list totaling 4357 structures. It is noted that some of the structures contained in other high profile PFAS lists, such as that provided by the OECD (OECD-PFAS 2018; “OECD: Comprehensive Global Database of PFASs”), were not contained in this initial PFASSTRUCTv1 list. This list served as a starting point for procuring the sample library of PFAS with which the EPA research effort could be undertaken (Patlewicz et al., 2019). This initial set of filters was retired and replaced with sets of substructural filters more closely aligned with EPA’s programmatic PFAS definitions; hence, the PFASSTRUCTv1 list has not been updated with new content since its initial release. However, the version released in March 2018 remains online, as originally defined, for historical reference. The various iterations of the PFASSTRUCT list available on the dashboard are clarified in the description of the PFASSTRUCT Navigation Panel list (https://comptox.epa.gov/dashboard/chemical-lists/PFASSTRUCT) and later iterations will be added into the same list.

Based on feedback within the Agency regarding the first released list, a second iteration (PFASSTRUCTV2 2021) was assembled using the OPPT TSCA, 2021 substructure filter RCF2CFR’R″ (R cannot be H). This substructure filter was applied to the updated DSSTox inventory resulting in the set of chemicals comprising PFASSTRUCTv2, released in November 2019; the resulting list contained a total of 6648 structures. The growth from the first list (4357 structures) to the newly defined substructure list primarily resulted from a dedicated effort to harvest additional PFAS chemicals from international regulatory lists, agency documentation, and peer-reviewed literature rather than from the new filter definition. The average number of new chemicals released every 6 months via the Dashboard was ca. 20,000. The increase of ~2300 PFAS chemicals, even with application of the new substructure filter, implies approximately 4% of the DSSTox database growth over this time was derived from PFAS structure harvesting alone. This second PFASSTRUCTv2 list likewise remains online in the form originally released to ensure access to the list for historical purposes.

**FIGURE 1 |:**
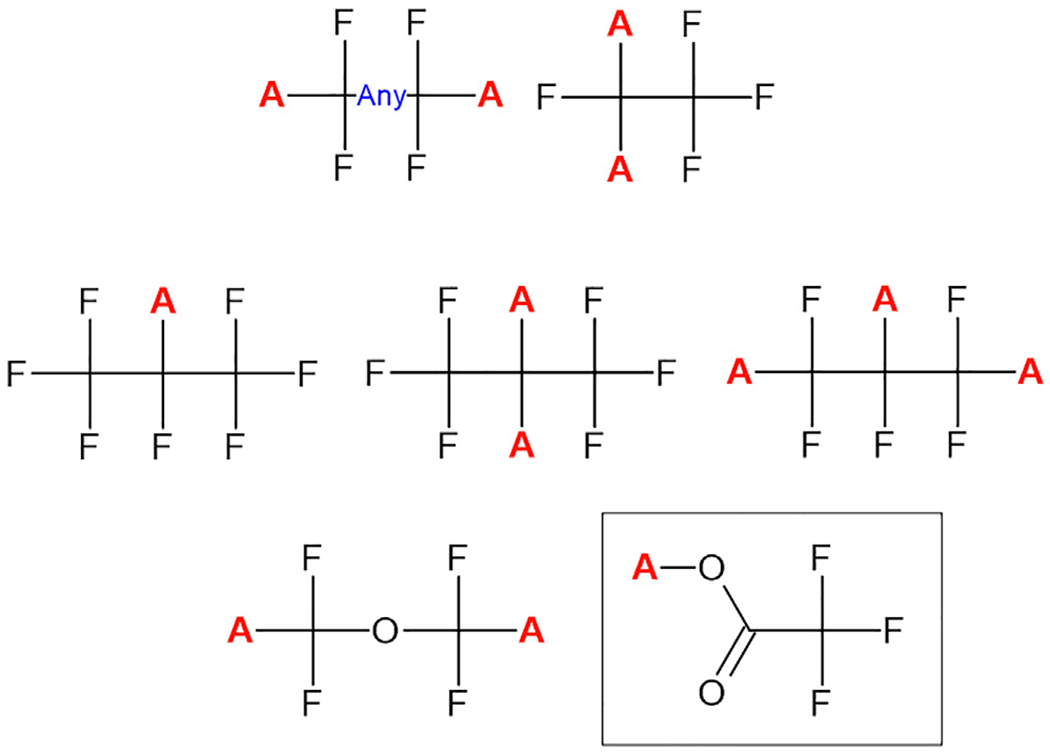
The collection of substructures used to define the PFASSTRUCTv3 list. Atoms replacing hydrogen (denoted by the red “A”) are all potential sites of substitution. The trifluoromethanesulfonic acid substructure (contained in the box) was included in PFASSTRUCTv3 but excluded in the subsequent PFASSTRUCTv4 iteration.

The third iteration of the PFASSTRUCT list departed from the substructural definition utilized for PFASSTRUCTv2, since specific substructures noted while aggregating chemicals from PFAS related databases, reports and literature, originally excluded from both lists 1 and 2, were later deemed by OPPT to be PFAS in nature. The new set of 7 substructural filters are shown in Figure 1, where all missing protons (with the red “A” denoting any substituent) in the substructures shown are substitution points. By way of example, EPA deemed trifluoroacetic acid (TFA) to be a PFAS chemical and, since it can be released from many substances via a hydrolysis reaction, the TFA substructural moiety was included as a substructure. This simple subjective addition added >60 chemicals to the list.).

As a result of the ongoing aggregation of PFAS chemicals from public sources, and the expansion of the substructure filters list, the number of chemicals in PFASSTRUCTv3 expanded to 8163 chemicals, almost doubling the number of chemicals contained in PFASSTRUCTv1. This third version is available online for reference (PFASSTRUCTV3 2021).

The fourth iteration of the PFASSTRUCT list, released in November 2021, was generated from all structural content available at the time of this most recent release (~906k substances) and contains a total of 10,776 chemicals. The substructural filters for this latest list differ from the previous v3 only by a slight adjustment: removal of the TFA moiety. This action resulted in all substances that contained TFA as a substructure, as a component of a mixture, or as a TFA salt, being removed. The original inclusion of TFA was as an ultrashort chain PFAS, but EPA’s OPPT deemed this moiety too short for inclusion in the PFAS definition.

### Per- and Polyfluoroalkyl Substances Without Explicit Structures

**FIGURE 2 |:**
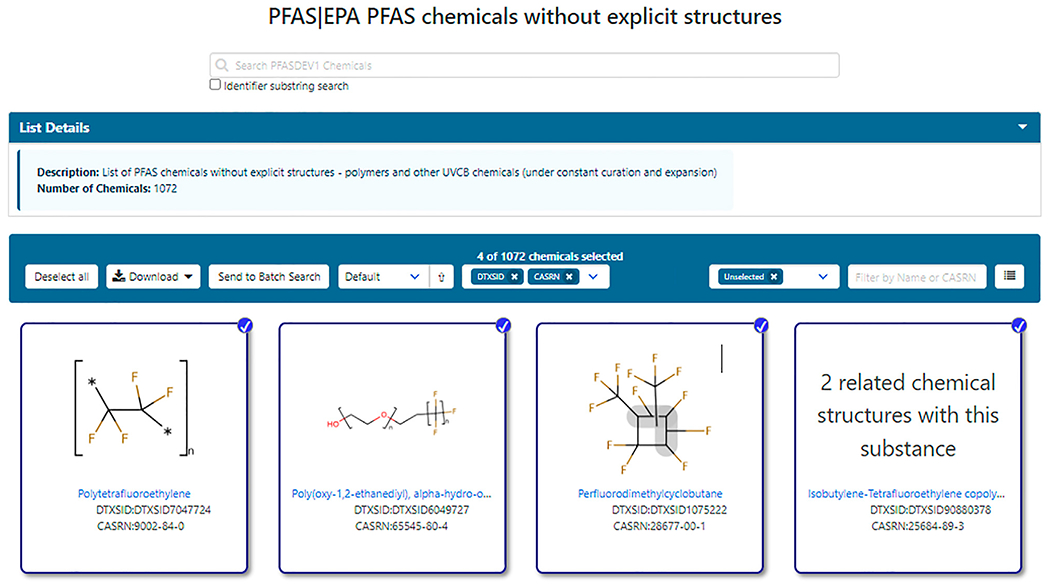
The list of non-explicit PFAS structures includes both Class 1 Markush structure representations as well as Class 2 which have no associated structures, but which may be mapped to related substances such as monomer units as exemplified by polytetrafluoroethylene.

In addition to structure-based lists, hundreds of PFAS chemicals without explicit structures, such as polymers, mixtures and ill-defined substances, that are associated with authoritative public lists (such as EPA and OECD) have been registered in DSSTox. Often referred to as UVCB (Unknown or Variable Composition, Complex Reaction Products and Biological Materials) substances, these can be divided into those substances amenable to representation in Markush form (such as some polymers and substances with variable chain lengths or indefinite substitution position–denoted here as Class 1) and those unamenable to structure definition (such as tars, oils, etc., denoted here as Class 2). An initial listing of such substances deemed to be PFAS, by virtue of their inclusion in public PFAS listings, was incorporated as part of the initial PFASMASTER list and consisting of the non-structural portions of the merged public PFAS lists. Subsequently, the unstructurable PFAS list was expanded by searching for chemicals in the larger DSSTox database using a set of name identifier substrings: perfluoro, polyfluoro, fluoroethylene, fluoropropylene, fluorobutene, fluoropolymer, “ethene, 1,1,2,2-tetrafluoro” (the PTFE monomer unit), chlorotrifluoroethylene, difluoromethylene, vinyl fluoride, tetrafluoro, pentafluoro, hexafluoro, heptafluoro, octafluoro, nonafluoro, decafluoro, and dodecafluoro. All resulting substances retrieved were then filtered to remove explicit chemical structures. The set of non-structurable chemicals classified as PFAS was published as a separate list, PFASDEV1 (https://comptox.epa.gov/dashboard/chemical_lists/PFASDEV1), and has been updated with each release of the Dashboard; it remains under constant curation and expansion. The list is composed of both Class 1 Markush structures and Class 2 UVCBs, which may have unknown or variable compositions or comprise a complex molecular combination or output from a chemical reaction. PFAS that are annotated with Markush structures during curation (Class 1) are also separately published as a list titled “EPAPFASCAT” (EPAPFASCAT 2021), and currently containing 326 entries in an internal version of the Dashboard, to be released publicly in 2022. Figure 2 shows a sample listing of members of the PFASDEV1 list.

## RESULTS

The four structure lists outlined above in the methods section illustrate several challenges faced in creating a definitive PFAS list: 1) recognizing that such a list, in order to be reproducible and transparent, must be structure-based; 2) deciding what structure-based rules and filters to use; and 3) recognizing that different regulatory and research needs may require more or less stringent structure-based filters. When based on clear structure-based rules, inclusion or exclusion from the PFAS group is entirely determined by and does not depend on any other factor except the structure itself. The common denominator of the various PFAS list filters and definitions presented thus far is that each results in a large number of diverse compounds being considered PFAS. Definitions can include straight chain polymers, polymers with side chains, and non-polymers. Compounds with no functional groups, containing only carbon and fluorine, are included in some lists, and compounds with a diverse set of functional groups are also included. And, whereas the name PFAS, per- and polyfluoroalkyl-substances, implies an alkyl substance, aromatic ring systems, including complex heterocyclics, can also be included in some definitions if they have a fluorinated alkyl side group.

Whereas it would be easy to run a simple substructure search against either a commercial database, such as CAS Scifinder (Scifinder 2021) or publicly available databases, such as PubChem (PubChem 2021) or ChemSpider (ChemSpider 2021), there are many potential issues with these results, including reliability of the source and relevance of the results to real-world, environmental exposure concerns. For example, although a PubChem search for the substructure CF2CF returns >337,000 hits (PubChem_CF2CF 2021) (reported on 12/12/2021), the majority of these chemicals do not have associated CASRNs listed in PubChem. PubChem includes large numbers of chemicals (hundreds of thousands) from on-demand chemical suppliers and virtual libraries, i.e., chemicals that do not exist in fact, at least yet. Supplier on-demand chemicals, and chemicals reported only in the chemistry synthesis literature, in virtual libraries, or in patents are unlikely to be of relevance for environmental study.

Chemical names alone are insufficient for identifying PFAS compounds in the absence of chemical structure. Chemicals are often included in databases and literature under non-systematic trade names, and the associated chemical structures can only be determined by referring to an external source of structural data. In contrast, systematic names (i.e., IUPAC or CAS Index Names) can be converted to structures using name-to-structure software, either commercial software products (e.g., ACD/Labs Name-to-Structure software (ACD/Labs 2021) used in our research) or open-source software (e.g., OPSIN, also used in our research (Lowe et al., 2011)). PFAS are routinely referred to by their common names; while some clearly indicate a compound as a PFAS (e.g., perfluorooctanesulfonic acid or perfluorooctanoic acid), many do not, especially in the common abbreviated forms (e.g., PFOS, PFOA or GenX). Also, while commonly perfluorooctanoic acid is considered to be one structure, mainly the linear form, the name itself does not specify the specific configuration and could apply to the 40 different structural isomers. This is similar for other common names of PFAS. Furthermore, because of the varying definitions of PFAS, even a systematic name would not necessarily indicate whether a compound is a PFAS, as only the structure and the associated definition of a PFAS define membership in the class. Hence, we posit that definitions of PFAS that are intended to be associated with a definitive and reproducible set of PFAS compounds should be based on chemical structure.

In 2021, the OECD adopted the broadest definition of PFAS yet proposed, only requiring one perfluorinated carbon moiety (i.e., –CF2–) and not limiting the structure as a whole to being aliphatic (OECD-PFAS 2021). Using this OECD definition to search the 906,511 substances in the latest public-facing Dashboard release (November 2021) identifies 38,382 PFAS. If, on the other hand, a terminal, fully fluorinated carbon is deemed to be the limiting substructure, then 32,940 PFAS are identified. If we apply the definition of Buck et al. (Buck et al., 2011) and require the entire structure to be aliphatic in nature, only 13,538 structures are identified (listed as “Buck text definition” in Table 1). Using this same aliphatic restriction but using Buck et al.’s definition of a terminal fully fluorinated carbon, there are 10,495 structures (listed as “Buck moiety definition” in Table 1). Using the original, more focused OECD Global PFAS list definition (OECD-PFAS 2018), there are 5,894 PFAS chemicals identifiable in the Dashboard (note this is nearly 2000 more PFAS than were included in the original OECD list). The EPA TSCA 2021 definition results in 9,389 chemicals identifiable in the Dashboard. Finally, using the PFASSTRUCv4 structure definition results in 10,776 chemicals. These lists are provided in the Supplementary Information.

The latest PFASSTRUCv4 definition yields 10,776 chemicals identifiable in the current version of the Dashboard. This definition can be narrowed even further to remove ions, radicals, and multicomponent structures (salts and mixtures). This results in 9,269 chemicals identifiable. The TSCA 2021 definition was also recently narrowed to remove ions, radicals, and multicomponent structures. This resulted in 7,950 chemicals being identified.

In order to compare what structures might be missed by the various PFAS definitions, the Dashboard was searched for all perfluorinated carbons or aliphatic structures consisting only of carbon and fluorine. There are 49 structures that meet that definition. Similarly, the Dashboard was searched for structures containing at least two fluorine atoms attached to a carbon atom but where the two fluorine atoms were not necessarily attached to the same carbon and with the only other elements being other halogens (bromine, chlorine, and iodine) and additional carbon atoms. No other restrictions were put on this search that resulted in 688 structures.

In the assembly of the data set, a check was made of the chemicals to determine their presence in different chemical lists by pushing the entire list of associated DTXSIDs for the PFASSTRUCTV4 list (PFASSTRUCTV4, 2021) to the batch search (Lowe and Williams 2021) and selecting lists deemed to be of interest. These are listed in Table 1.

For preparation and comparison of subsets, generated lists were imported into SAS version 9.4 (TS1M1) (SAS Institute, Cary, NC) and compared. Structures were compared using the Chemicals Dashboard’s DTXSID.

## DISCUSSION

The specific chemical list collections associated with this publication are available online for download (Dashboard_Downloads 2021). Following each update of the Dashboard release, a subset of these lists is updated and made available to the community to source and reuse for their own purposes. The definition of a specific list can be context sensitive. For example, the subjective decision to remove certain chemicals (e.g., non-charge-balanced chemicals such as bare anions, etc.) can be deemed appropriate because such chemicals cannot be acquired commercially, whereas inclusion of such chemicals might be considered appropriate when considering results of environmental samples analyzed by mass spectroscopy.

The OECD 2021 list having the least restrictive substructure definition is the most fully encompassing, with all other lists being subsets of OECD-PFAS 2021. The exception to this is the Perhalocarbons (PHC) list that contains structures with a minimum of 2 fluorine atoms and additionally only C, Br, Cl, or I in the formula. There are 73 structures on the PHC list that are not included in the OECD-PFAS 2021 list. These are either aromatic with no aliphatic portion or they contain multiple other halogens in the structures and no two fluorine atoms attached to the same carbon and, therefore, fall outside of the OECD definition. The Buck text list searches for the same moiety as the OECD-PFAS 2021 list, but the difference of 24,844 structures on the OECD-PFAS 2021 list that are not on the Buck text list indicates the number of aromatic structures that are eliminated. Similarly, when searching on the terminal -CF3 moiety, there is a difference of 22,445 structures compared to when aromatics are included and when they are not.

**FIGURE 3 |:**
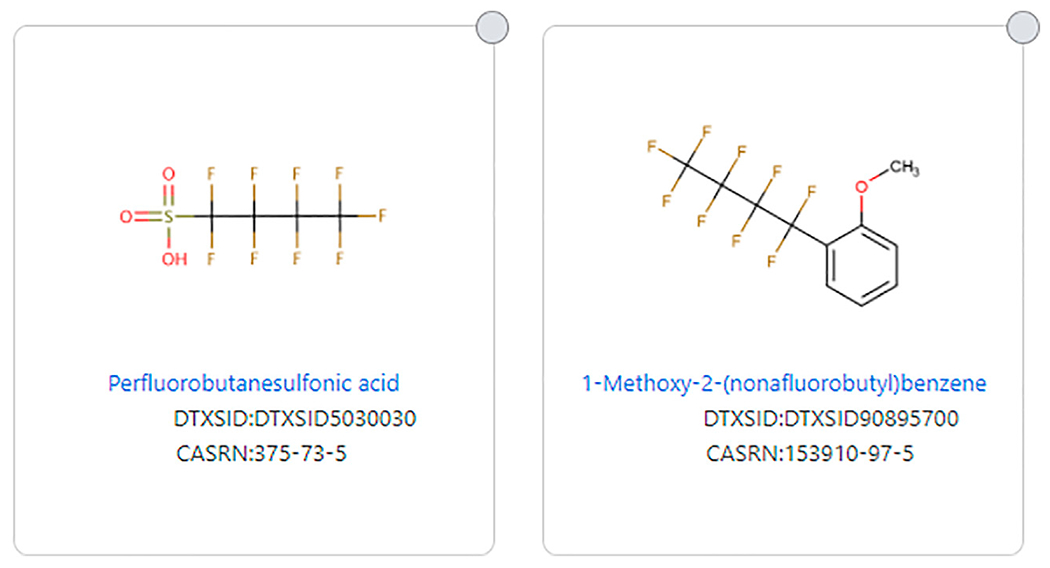
Example of structures that differ by being fully aliphatic or only partially aliphatic with an aromatic substituent.

**FIGURE 4 |:**
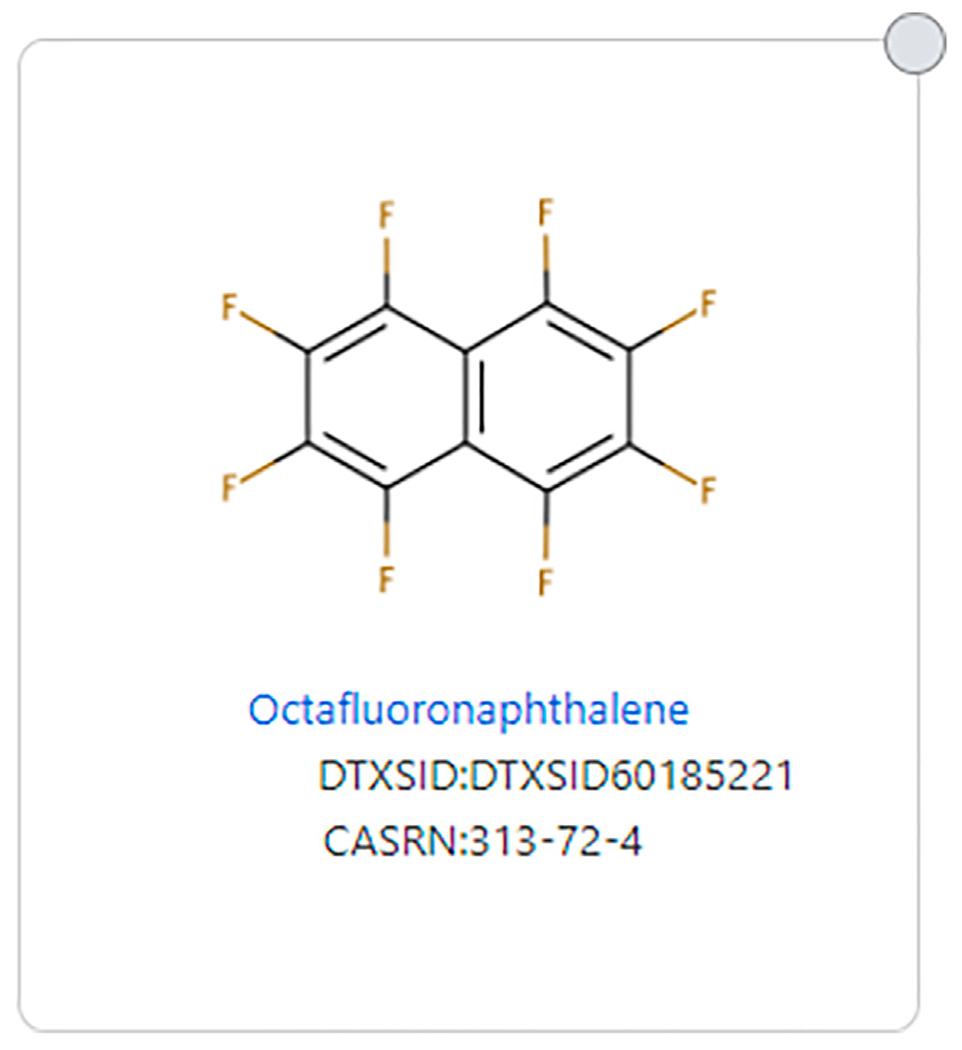
Example of a fully fluorinated and aromatic structure that does not meet any PFAS definition.

The issue of aromatics is important as two structures can have the exact same fluorinated substructure, but if one has an aromatic substructure in the non-fluorinated portion, it would not be considered a PFAS by the original PFAS definitions. For example, Perfluorobutanesulfonic acid (DTXSID5030030) fits all definitions of PFAS, but 1-methoxy-2-(nonafluorobutyl)benzene (DTXSID90895700), which has the same fluorinated portion, does not fit all PFAS definitions due to its aromatic substructure (see Figure 3). Wang et al. (OECD-PFAS 2021) discusses this issue and reasoning for allowing aromatics as long as the -CF2- moiety is aliphatic. Some structures that consist only of carbon and fluorine do not meet any definition of PFAS because there is no aliphatic portion of the structure, such as octafluoronaphthalene (DTXSID60185221) (See Figure 4).

**FIGURE 5 |:**
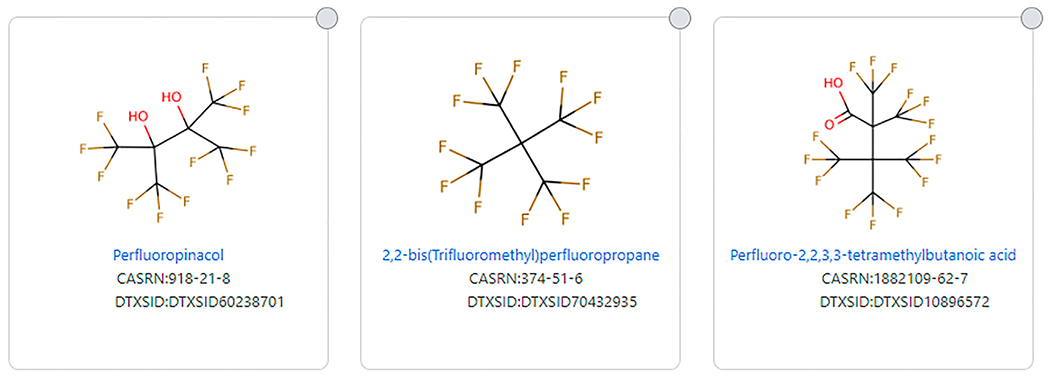
Examples of highly branched structures that do not fit the EPA TSCA 2021 PFAS substructure definition.

OECD, in their 2018 focus list, attempted to narrow that study for PFAS with a more restricted definition, as discussed above. EPA TSCA 2021 and the PFASSTRUC list also attempt to narrow the definition of a PFAS. There can be a variety of reasons for doing so, but caution is warranted when narrowing the definition in that chemicals may be eliminated that are not intended to be eliminated. For example, the EPA TSCA 2021 definition eliminates several chemicals that “most” would say are PFAS, but the structures are so highly branched, the definition is not met because two fluorinated carbons do not occur side by side. Examples include 2,2-bis (Trifluoromethyl) perfluoropropane (DTXSID70432935), Perfluoropinacol (DTXSID60238701), and 4,4,4-Trifluoro-2,2,3,3-tetrakis (trifluoromethyl)butanoic acid (DTXSID10896572), the latter being a highly branched structural isomer of perfluorooctanoic acid (DTXSID8031865) (**See** Figure 5).

**FIGURE 6 |:**
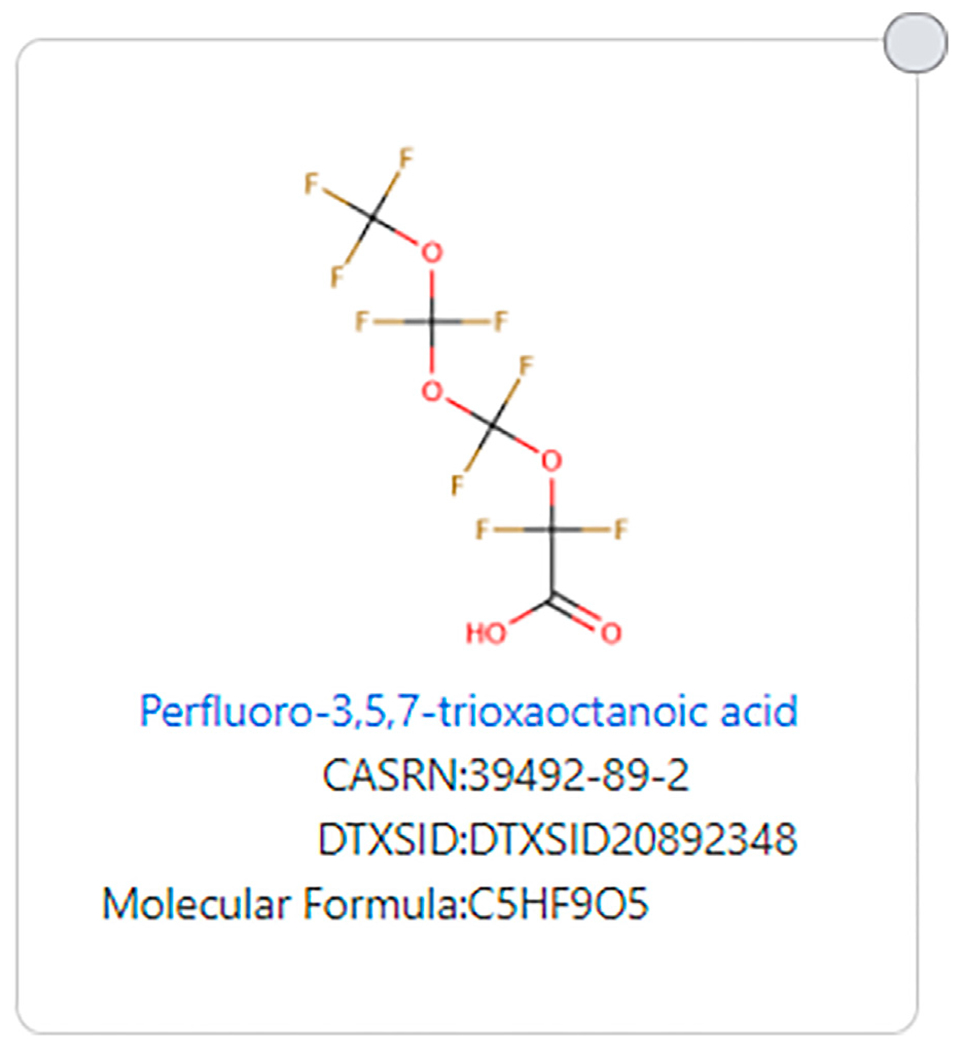
Example of a PFAS ether that does not fit the EPA TSCA 2021 PFAS substructure definition.

Similarly, structures with many ether groups also do not meet the EPA TSCA 2021 definition, an example being perfluoro-3,5,7-trioxaoctanoic acid (DTXSID20892348, see Figure 6).

**FIGURE 7 |:**
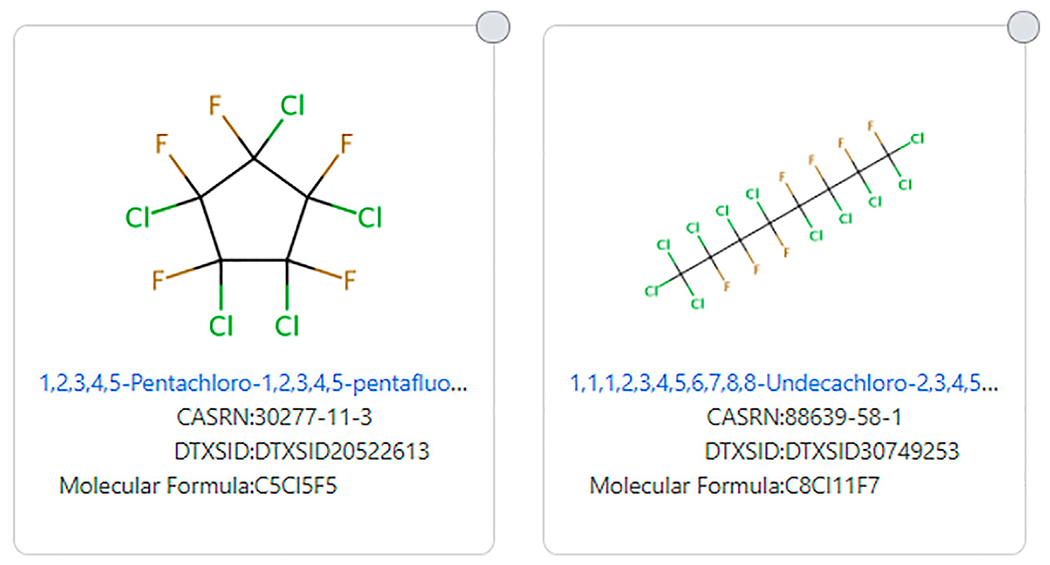
Examples of halogenated chains that do not fit any PFAS structure definition.

Other halogens can also eliminate chemicals from being called a PFAS from even the most encompassing definitions of PFAS, such as OECD-PFAS 2021. Some of the structures that are excluded from all existing PFAS definitions include 1,1,1,2,3,4,5,6,7,8,8-Undecachloro-2,3,4,5,6,7,8-heptafluorooctane (DTXSID30749253) and 1,2,3,4,5-Pentachloro-1,2,3,4,5-pentafluorocyclopentane (DTXSID20522613). These structures are shown in Figure 7.

**FIGURE 8 |:**
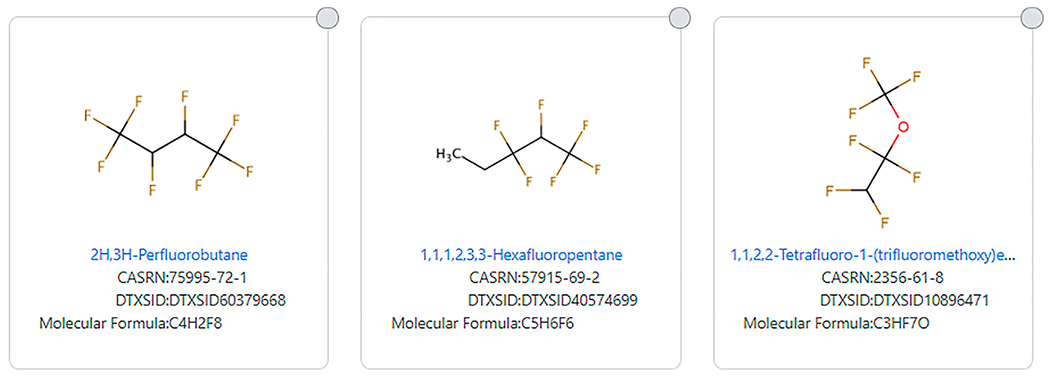
Examples of highly fluorinated chains that do not fit the EPA TSCA 2021 PFAS substructure definition.

The TSCA 2021 substructure also narrows the definition by not allowing a hydrogen atom to replace any of the R groups attached to the defined substructure. This eliminates structures that meet other definitions of PFAS. Examples include 1,1,1,2,3,3-Hexafluoropentane (DTXSID40574699) and 2H, 3H-Perfluorobutane (DTXSID60379668). 1,1,2,2-Tetrafluoro-1-(trifluoromethoxy)ethane (DTXSID10896471) is eliminated from the EPA TSCA 2021 definition as a result of the attached hydrogen atom attached to the fluorinated carbon as well as the presence of an ether group in the third example depicted in Figure 8.

**FIGURE 9 |:**
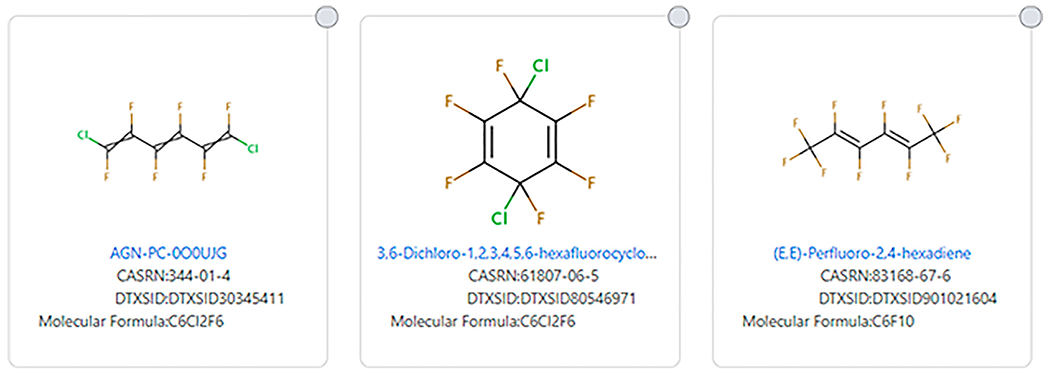
Examples of alkenic fluorinated chain and ring systems that do not fit the EPA TSCA 2021 PFAS substructure definition. The first two do not fit any PFAS structural definition.

Double and triple bonds can also complicate which structures are considered PFAS (see Figure 9 for examples). (E,E)-Perfluoro-2,4-hexadiene (DTXSID901021604) meets many definitions of PFAS, but it does not meet the EPA TSCA 2021 definition of a PFAS. However, 1,6-Dichloro-1,2,3,4,5,6-hexafluorohexa-1,3,5-triene (DTXSID30345411) and 3,6-Dichloro-1,2,3,4,5,6-hexafluorocyclohexa-1,4-diene (DTXSID80546971) do not meet any definition of PFAS, but they have similarities to some PFAS.

**FIGURE 10 |:**
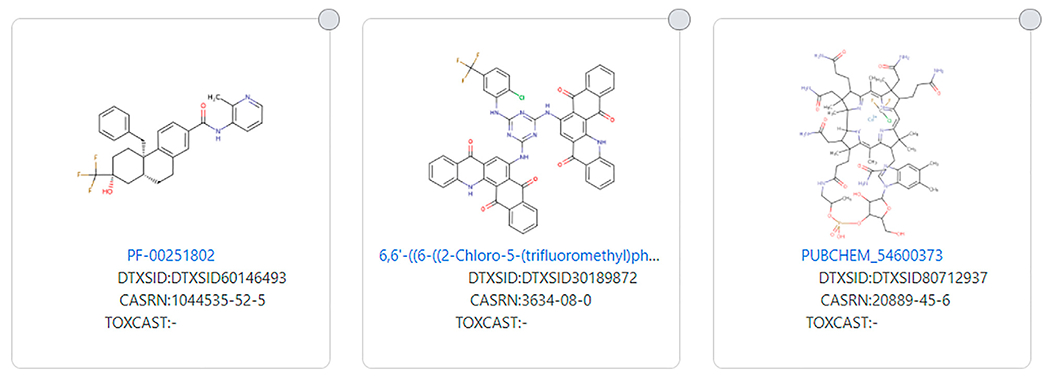
Examples of large molecules with a very small fluorinated moiety that fits the OECD-PFAS 2021 definition but not more restricted definitions.

As stated, the EPA TSCA 2021 and Dashboard definitions attempt to narrow the PFAS definition and this can result in structures that do not fit the PFAS definition that many might consider to be a PFAS. Conversely, the opposite is true with a wider definition such as used by OECD-PFAS 2021. Several structures fit the OECD-PFAS 2021 definition, but the fluorinated portion of the molecule is only a tiny part of the molecule, molecular weight wise. Examples of this include DTXSID80712937 and DTXSID30189872 (see Figure 10). Many investigated and marketed medications fit this wide definition of PFAS, and whereas the fluorinated portion of the molecule may be important function-wise, it constitutes only a small portion of the entire structure. An example of this is an investigated medication PF-00251802 (DTXSID60146493) (see Figure 10).

The OECD 2021 definition is expansive and includes almost all structures that could possibly be considered a PFAS, with the potential exceptions noted previously. Conversely, the expansive definition includes structures that may or may not be considered a PFAS by the scientific community, and the PFAS portion may be the least important part of the compound from an environmental contamination or toxicity perspective. The TSCA 2021 and PFASSTRUCT definitions attempt to narrow the PFAS definition to focus the list to what is more important for EPA programmatic purposes. However, the structural restrictions may or may not fulfill the intended purpose of narrowing the list. The structural restriction may also create a “loophole” that filters out a desired structure.

Because all the PFAS definitions presented here are based on structure filters and physicochemical or toxicological properties were not considered, the resulting PFAS will have a wide variety of physicochemical or toxicological properties. Some PFAS may have properties that are more similar to non-PFAS chemicals than to most PFAS. For example, PFAS that consists entirely of carbon and fluorine will have more in common with non-PFAS chemicals consisting entirely of carbon, fluorine, and chlorine than with most other PFAS. Thus, when creating a PFAS definition, the division between PFAS and non-PFAS may be necessarily arbitrary, and the reason for the definition needs to be considered.

## FUTURE WORK

The extraction, curation and assembly of data associated with PFAS chemicals will continue unabated as new chemicals are reported in the literature, in regulatory lists and other sources. This will mean that there will likely be an updated PFASSTRUCT list released with each future release of the Dashboard. The manner by which the lists are assembled may also change in future iterations based on EPA programmatic needs and different contexts. The continued expansion of the PFAS data collection will benefit from our efforts to develop categorization approaches (Patlewicz et al., 2019). The originally developed 112 categories represented as Markush structures (EPAPFASCAT 2021)has expanded to over 320 in total and efforts will continue to expand on this categorization effort using this approach. We are also considering how automated taxonomic based categorization, as enabled by tools such as ClassyFire (Djoumbou Feunang et al., 2016), can provide an additional categorization approach. Our efforts to develop software approaches to identify branching in PFAS chains are represented in this Special Issue (Richard et al., 2022).

The PFAS lists discussed in this work are valuable to support many of research efforts within the EPA by providing a clear structure-bounded PFAS landscape of interest in each case. They have been used to inform the selection of chemicals for our ongoing *in vitro* bioactivity studies, as well as to support EPA’s non-targeted analysis mass spectrometry studies (Newton et al., 2018; Ulrich et al., 2018; Sobus et al., 2019) and automated and comprehensive non-targeted analysis PFAS annotation (Koelmel et al., 2021). The lists have also proven to be pivotal for the EPA’s National Testing Strategy (PFAS_Roadmap 2021) as a starting point to filter down to a list of PFAS from which potential candidates for test orders could be identified as part of a structural categorization approach. The PFASSTRUCT list formed the “PFAS landscape” of interest from which categorization approaches could be used to segment the landscape and facilitate the identification of representative members to characterize each category. Potential candidates for test orders focused on those structural categories that were data poor in terms of their hazard data. Further work will explore how structural categories can be informed by bioactivity and physicochemical data to define categories of PFAS that are similar by various contexts. A manuscript is presently in preparation describing the assembly of a PFAS list, and associated categorization of that list, to provide a foundational dataset that has been used as a basis to select chemicals for the EPA’s PFAS National Testing Strategy effort presently underway.

## CONCLUSION

The EPA has been aggregating and curating data and information about PFAS chemicals to support ongoing research efforts into the properties and toxicity of this class of chemicals. A single and clear definition and community consensus regarding what is a PFAS currently does not exist. That the acronym PFAS is near-universally understood to represent “per- AND polyfluoroalkyl substances” (i.e., where polyfluoro implies 2 or more alkyl fluorines anywhere in the molecule) is by any reasonable measure overly broad, lending itself to multiple, application-specific definitions such as those presented in this paper. Additionally, and primarily for historical reasons, the term PFAS explicitly includes the term “alkyl,” whereas there is insufficient scientific rationale for excluding compounds in which an aromatic system is separated from a per (or poly) fluoro alkyl chain capable of degrading to a compound of concern, such as PFOA. Elsewhere in this journal issue, Richard et al. (2022) present a computational approach to detect a terminal perfluoroheptyl group bonded to carbon (C7F15-C), which is assumed to potentially confer the ability to degrade to PFOA irrespective of other moieties present in the molecule (such as an aromatic system). The computational approach is a means to aid the PFAS community in interpreting which chemicals fall under the Conference of the Parties to the Stockholm Convention on Persistent Organic Pollutants’ 2021 Indicative listing of “PFOA, its salts and related compounds” for potential regulatory consideration (Stockholm_Convention 2021).

Structural definitions of PFAS space have the advantage of being clear, reproducible, chemically intuitive, and computationally exacting. However, these definitions act primarily as conceptual surrogates, helping us to structurally bound the PFAS chemical universe to compounds that one might reasonably assume can exhibit “PFAS-like behavior.” This latter term, however, is also vague and problematic in that it is anchored both to property characteristics that have led to widespread use and release of PFAS compounds, as well as to concerns for bioaccumulation and toxicity. These two types of properties derive from underlying chemistry of the class and, thus, are entangled. And whereas uses of PFAS are extensive, toxicity data are available for a relatively small number of well- studied PFAS, such as PFOA and PFOS. Hence, structure definitions of PFAS, while exceedingly useful in providing bounded chemical spaces, are ultimately limited, should be tailored to the problem at hand, and should not be fixed in stone.

As part of our own research, and to support our efforts to disseminate data to the community, we have curated, compiled and published several “PFAS lists” that have been made available to the community via the publicly accessible CompTox Chemicals Dashboard. Finally, the role of quality DSSTox curation, structure-annotation, and aggregation of a range of publicly available PFAS compound listings cannot be overstated. Chemical structures provide inputs for modeling to predict physicochemical properties, fate and transport, and biological activities and toxicity. This publication has provided an overview of our efforts to date to deliver (sub) structural based definitions of PFAS, including the latest definitions from OECD, as well as an approach to assemble a list of UVCB non-structurable PFAS chemicals. We have also critically examined the ways in which these varied definitions are either too broad or limiting. Despite these caveats, the approaches and PFAS structure-annotated lists described herein, along with the associated data and property linkages accessible through the Dashboard, provide a strong foundation to support PFAS research efforts presently underway within the EPA, as well as across the international scientific community.

## Supplementary Material

Supplement1

## Figures and Tables

**TABLE 1 |:** Number of structures (#) contained within different list definitions based on the chemicals in the CompTox Chemicals Dashboard database release (December 2021). Some of these subset lists are contained in the supplementary information files.

List Name	Structural definition	# Structures
OECD 2021 contains -CF2-	Contains aliphatic and saturated -CF2-	38,382
OECD 2021 contains -CF3	Contains aliphatic and saturated -CF3	32,940
Buck text definition	Aliphatic structures containing -CF2-	13,538
Buck moiety definition	Aliphatic structures containing -CF3	10,495
OECD focus list 2018	–CnF2n–, n ≥ 3 or –CnF2nOCmF2m–, n and m ≥ 1	5,894
TSCA 2021	R-(CF2)-C(F)(R′)R″; both CF2 and CF are saturated carbons; R, R′ or R″ cannot be H	9,389
TSCA 2021, no ions, radicals, or multicomponent structures	TSCA 2021 with ions, radicals, and multicomponent structures removed	7,950
PFASSTRUCTv3 filters	7 structures noted above	11,333
PFASSTRUCTv4 filters (no TFA)	6 structures noted above	10,776
PFASSTRUCTv4 filters (no TFA, ions, radicals, multicomponent structures)	Dashboard structures (no TFA, ions, radicals, multicomponent structures)	9,269
Alkyl Perfluorocarbons (containing C and F)	Structures containing only C and F (no double or triple bonds, no radicals)	49
Perhalocarbons (at least 2 fluorine atoms)	Structures containing at least 2 F and additionally only C, Br, CI, or I	688

## Data Availability

The original contributions presented in the study are included in the article/Supplementary Material, further inquiries can be directed to the corresponding author.

## Footnotes

1 GenX is more precisely a trade name for a chemical process to produce an alternative to a perfluorooctanoic acid (PFOA or C8) in the synthesis of PTFE (i.e., Teflon).

## References

[R1] ACD/Labs (2021). ‘ACD/Labs Nomenclature Software’. Available at: https://www.acdlabs.com/products/draw_nom/nom/name/ (Accessed 12 15, 2021).

[R2] Buck RC, Franklin J, Berger U, Conder JM, Cousins IT, de Voogt P, (2011). Perfluoroalkyl and Polyfluoroalkyl Substances in the Environment: Terminology, Classification, and Origins. Integr. Environ. Assess. Manag 7, 513–541. doi:10.1002/ieam.258 21793199 PMC3214619

[R3] ChemSpider (2021). Available at: http://www.chemspider.com/.

[R4] Dashboard_Downloads (2021). ‘CompTox Chemicals Dashboard: Downloads Page’. Available at: https://comptox.epa.gov/dashboard/downloads (Accessed 12 15, 2021).

[R5] Dashboard_Lists (2021). ‘Comptox Chemicals Dashboard List of Lists of Chemicals’. Available at: https://comptox.epa.gov/dashboard/chemical-lists (Accessed 12 15, 2021).

[R6] Djoumbou Feunang Y, Eisner R, Knox C, Chepelev L, Hastings J, Owen G, (2016). ClassyFire: Automated Chemical Classification with a Comprehensive, Computable Taxonomy. J. Cheminform 8, 61. doi:10.1186/s13321-016-0174-y 27867422 PMC5096306

[R7] Drinking Water Contaminant Candidate List 5-Draft (2021). ‘Drinking Water Contaminant Candidate List 5-Draft’. Available at: https://www.regulations.gov/document/EPA-HQ-OW-2018-0594-0031 (Accessed 12 29, 2021).

[R8] Epa Tsca (2021). EPA’s Per- and Polyfluoroalkyl Substances (PFAS) Action Plan’. Available at: https://www.epa.gov/sites/default/files/2019-02/documents/pfas_action_plan_021319_508compliant_1.pdf (Accessed 12 15, 2021).

[R9] EPAPFASCAT. (2021). ‘CompTox Chemicals Dashboard List: PFAS|EPA Structure-Based Categories’. Available at: https://comptox.epa.gov/dashboard/chemical-lists/EPAPFASCAT (Accessed 12 15, 2021).

[R10] Fourches D, Muratov E, and Tropsha A (2010). Trust, but Verify: On the Importance of Chemical Structure Curation in Cheminformatics and QSAR Modeling Research. J. Chem. Inf. Model 50, 1189–1204. doi:10.1021/ci100176x 20572635 PMC2989419

[R11] Glüge J, Scheringer M, Cousins IT, DeWitt JC, Goldenman G, Herzke D, (2020). An Overview of the Uses of Per- and Polyfluoroalkyl Substances (PFAS). Environ. Sci. Process. Impacts 22, 2345–2373. doi:10.1039/d0em00291g 33125022 PMC7784712

[R12] Grulke CM, Williams AJ, Thillanadarajah I, and Richard AM (2019). EPA’s DSSTox Database: History of Development of a Curated Chemistry Resource Supporting Computational Toxicology Research. Comput. Toxicol 12, 100096. doi:10.1016/j.comtox.2019.100096 PMC7787967 33426407

[R13] Kavlock R, Chandler K, Houck K, Hunter S, Judson R, Kleinstreuer N, (2012). Update on EPA’s ToxCast Program: Providing High Throughput Decision Support Tools for Chemical Risk Management. Chem. Res. Toxicol 25, 1287–1302. doi:10.1021/tx3000939 22519603

[R14] Koelmel JP, Stelben P, McDonough CA, Dukes DA, Aristizabal-Henao JJ, Nason SL, (2021). ‘Fluoro Match 2.0-making Automated and Comprehensive Non-targeted PFAS Annotation a Reality’. Anal. Bioanal. Chem doi:10.1007/s00216-021-03392-7 PMC10228292 34014358

[R15] List_Pesticide_Packaging (2021). ‘CompTox Chemicals Dashboard List: PFAS|EPA PFAS Substances in Pesticide Packaging’. Available at: https://comptox.epa.gov/dashboard/chemical-lists/PFASPACKAGING (Accessed 12 15, 2021).

[R16] Lowe CN, and Williams AJ (2021). Enabling High-Throughput Searches for Multiple Chemical Data Using the U.S.-EPA CompTox Chemicals Dashboard. J. Chem. Inf. Model 61, 565–570. doi:10.1021/acs.jcim.0c01273 33481596 PMC8630643

[R17] Lowe DM, Corbett PT, Murray-Rust P, and Glen RC (2011). Chemical Name to Structure: OPSIN, an Open Source Solution. J. Chem. Inf. Model 51, 739–753. doi:10.1021/ci100384d 21384929

[R18] National Defense Authorization (2020). National Defense Authorization Act for Fiscal Year. Available at: https://www.congress.gov/bill/116th-congress/senate-bill/1790 (Accessed 12 15, 2021).

[R19] Newton SR, McMahen RL, Sobus JR, Mansouri K, Williams AJ, McEachran AD, (2018). Suspect Screening and Non-targeted Analysis of Drinking Water Using point-of-use Filters. Environ. Pollut 234, 297–306. doi:10.1016/j.envpol.2017.11.033 29182974 PMC6145080

[R20] OECD (2021). ‘OPSIN Comprehensive Global Database of PFASs’. Available at: https://www.oecd.org/chemicalsafety/portal-perfluorinated-chemicals/ (Accessed 12 15, 2021).

[R21] OECD-PFAS. (2018). ‘OECD, Summary Report on Updating the OECD 2007 List of Per- and Polyfluorinated Substances (PFASs), Report ENV/JM/MONO. Available at: http://www.oecd.org/officialdocuments/publicdisplaydocumentpdf/?cote=ENV-JM-MONO(2018)7&doclanguage=en (Accessed 12 15, 2021).7.

[R22] OECD-PFAS. (2021). ‘Organization for Economic Co-operation and Development (OECD). 2021. Reconciling Terminology of the Universe of Per- and Polyfluoroalkyl Substances: Recommendations and Practical Guidance. Series on Risk Management No. 61. ’. Available at: https://www.oecd.org/chemicalsafety/portal-perfluorinated-chemicals/terminology-per-and-polyfluoroalkyl-substances.pdf (Accessed 12 15, 2021).

[R23] OPSIN. (2021). ‘OPSIN Application’. Available at: https://opsin.ch.cam.ac.uk/ (Accessed 12 15, 2021).

[R24] Patlewicz G, Richard AM, Williams AJ, Grulke CM, Sams R, Lambert J, (2019). A Chemical Category-Based Prioritization Approach for Selecting 75 Per- and Polyfluoroalkyl Substances (PFAS) for Tiered Toxicity and Toxicokinetic Testing. Environ. Health Perspect 127, 14501. doi:10.1289/EHP4555 30632786 PMC6378680

[R25] PFAS_Roadmap (2021). ‘PFAS Strategic Roadmap: EPA’s Commitments to Action 2021-2024’. Available at: https://www.epa.gov/pfas/pfas-strategic-roadmap-epas-commitments-action-2021-2024 (Accessed 12 15, 2021).

[R26] PFASSTRUCT_Navigation (2021). ‘CompTox Chemicals Dashboard: Navigation Panel to PFAS Structure Lists’. Available at: https://comptox.epa.gov/dashboard/chemical-lists/PFASSTRUCT (Accessed 12 15, 2021).

[R27] PFASSTRUCTV1 (2021). ‘CompTox Chemicals Dashboard List: PFAS|EPA: PFAS Structures in DSSTox (Update March 2018)’. Available at: https://comptox.epa.gov/dashboard/chemical_lists/PFASSTRUCTV1 (Accessed 12 15, 2021).

[R28] PFASSTRUCTV2 (2021). ‘CompTox Chemicals Dashboard List: PFAS|EPA: PFAS Structures in DSSTox (Update November 2019)’. Available at: https://comptox.epa.gov/dashboard/chemical-lists/PFASSTRUCTv2 (Accessed 12 15, 2021).

[R29] PFASSTRUCTV3 (2021). ‘CompTox Chemicals Dashboard List: PFAS|EPA: PFAS Structures in DSSTox (Update August 2020)’. Available at: https://comptox.epa.gov/dashboard/chemical-lists/PFASSTRUCTV3 (Accessed 12 15, 2021).

[R30] PubChem. (2021). ‘PubChem’. Available at: https://pubchem.ncbi.nlm.nih.gov/ (Accessed 12 07, 2021).

[R31] PubChem_CF2CF (2021). Search for the Substructure CF2CF. Available at: F&input_type=smarts&fullsearch=true&page=1 https://pubchem.ncbi.nlm.nih.gov/#query=C(CF)(F) (Accessed 12 15, 2021).

[R32] Richard AM, Hidle H, Patlewicz G, and Williams AJ (2022). Identification of Branched and Linear Forms of PFOA and Potential Precursors: A User-friendly SMILES Structure-based Approach. Front. Environ. Sci 10, 865488. doi:10.3389/fenvs.2022.865488 PMC9048161 35494535

[R33] Scifinder (2021). ‘Chemical Abstracts Service Scifinder’. Available at: https://scifinder.cas.org/ (Accessed 12 15, 2021).

[R34] Sobus JR, Grossman JN, Chao A, Singh R, Williams AJ, Grulke CM, (2019). Using Prepared Mixtures of ToxCast Chemicals to Evaluate Non-targeted Analysis (NTA) Method Performance. Anal. Bioanal. Chem 411, 835–851. doi:10.1007/s00216-018-1526-4 30612177 PMC6469933

[R35] Stockholm_Convention (2021). ‘Stockholm Convention on Persistent Organic Pollutants’ 2021 Indicative Listing of “PFOA, its Salts and Related Compounds”. Available at: http://chm.pops.int/Implementation/Alternatives/AlternativestoPOPs/ChemicalslistedinAnnexA/PFOA/tabid/8292/Default.aspx (Accessed 12 15, 2021).

[R36] TSCA Substances (2020). “Toxic Substances Control Act Reporting and Recordkeeping Requirements for Perfluoroalkyl and Polyfluoroalkyl Substances.” in.

[R37] Ulrich EM, Sobus JR, Grulke CM, Richard AM, Newton SR, Strynar MJ, (2018). EPA’s Non-targeted Analysis Collaborative Trial (ENTACT): Genesis, Design, and Initial Findings. Anal. Bioanal. Chem 411, 853–866. doi:10.1007/s00216-018-1435-6 30519961 PMC7477838

[R38] Williams AJ, Grulke CM, Edwards J, McEachran AD, Mansouri K, Baker NC, (2017). The CompTox Chemistry Dashboard: a Community Data Resource for Environmental Chemistry. J. Cheminform 9, 61. doi:10.1186/s13321-017-0247-6 29185060 PMC5705535

