# Supplementary material for: Assembly and Curation of Lists of Per- and Polyfluoroalkyl Substances (PFAS) to Support Environmental Science Research
Source: Front Environ Sci. Author manuscript; Available in PMC 2023 Apr 5. (PMC9350880; doi:10.3389/fenvs.2022.850019)
Supplement: Supplement1 [file NIHMS1824259-supplement-Supplement1.zip › DataSheet1_Assembly and Curation of Lists of Per- and Polyfluoroalkyl Substances (PFAS) to Support Environmental Science Research.docx]

**Supplementary Information 1: An Analysis of Data Gathering of PFAS Chemicals from Public Resources**

Significant numbers of PFAS, according to different substructural search criteria discussed in the paper, have been harvested or validated against public resources over the past ~5 years. Many websites have been sampled, including PubChem, ChemSpider and the more recent Common Chemistry website. As a smaller database with high reliability due to its sourcing from CAS, Common Chemistry has been used primarily for the validation of CASRNs in data obtained from other public resources; these other resources provide much larger samples but may rely partially or wholly on lower-quality depositor-supplied data. These resources are utilized with a combination of manual queries, batch exports, and programmatic access using application programming interfaces (APIs). Using this approach, subsets of data for registration into the DSSTox database have been sourced. For PubChem in particular, that availability of a rich API to query the data is useful to examine the growth in the number of PFAS chemicals contained within the resource. A number of examples representing both data harvesting and profiling of PFAS chemical datasets are listed below.

On December 26^th^, 2019, a query of PubChem (<https://pubchem.ncbi.nlm.nih.gov/>) using a substructural query of RCF2CFR’R” (see the main manuscript for the relevance of this substructure) was performed. 9,311 records containing this specific substructure were identified. PubChem CIDs and associated InChIKeys were then extracted for the 9,311 records. Approximately 4.3% of structures described by the harvested InChIKeys were present in DSSTox. Despite our interest in expanding the PFAS chemicals dataset to support our research, we are cautious that this expansion is not based simply on supplier-on-demand chemicals or harvesting of chemicals from patents that may simply be associated with an enumeration exercise of an ambiguous structural representation. As a proxy to validate the existence of chemicals, the InChIKeys which did not match an existing record in the DSSTox database (8,909 records) were then used to query ChemSpider (<http://www.chemspider.com/>) to obtain CASRN(s) if available. 758 (8.5%) of the queried InChIKeys were mapped to one or more CASRNs. This subset of chemicals was registered into the DSSTox database.

On November 8^th^, 2021, a more general substructural query for fluorinated chemicals was performed (RCF2CFR’R” with lone hydrogens permitted as R groups, i.e., SMARTS C(CF)(F)F), identifying 332,989 records (332,206 distinct InChIKeys). Depositor-supplied CASRNs were then extracted from PubChem for this list using the native API (<https://pubchemdocs.ncbi.nlm.nih.gov/programmatic-access>). Due to the major improvement in efficiency of the REST over the VIEW API, CASRNs were extracted from depositor-supplied synonyms rather than from PubChem’s CAS heading. In total, 11,446 (3.4%) of the results had an associated CASRN, with 204 of these having more than one CASRN. Registry numbers which did not match an existing record in DSSTox (3,094 records in total) were then validated against Common Chemistry (<https://commonchemistry.cas.org/>) via API, which discovered 11 matches (<0.4% of the hits). Those 11 chemical substances were then added to DSSTox as high priority data, and the remaining 3,083 queued for registration after curator review.

On December 13^th^, 2021, the same query was repeated, identifying 337,504 records (336,673 distinct InChIKeys). However, upon inspection, it became clear that the net increase of 4,467 structures over the month derived from the *removal* of 30,469 structures and then the *addition* of 34,936 others. These sets were non-overlapping, i.e., no structures were added again after removal. The removed structures were sourced from a batch registration of structures from Google Patents on August 12^th^ and 13^th^, 2021; all structures registered in this batch have subsequently been rendered non-live in PubChem by removal of their associated substance records the reason for which we are unsure. Of the ~35,000 substances added, only two had associated CASRNs. Neither of these substances existed in DSSTox, and neither of the two CASRNs could be validated in Common Chemistry. Both were added to the database for future reference.

This indicates that while there may be many tens, if not thousands, of PFAS-like chemicals flowing into public domain databases, only a fraction of these may be contained within the Scifinder database (i.e., with CASRNs). The source of these non-registered chemicals is expected to be from chemical vendor supplier-on-demand chemicals with theoretical syntheses, as well as from patent extraction, which uses chemical name entity recognition approaches and name-to-structure nomenclature conversion algorithms to generate chemical structures from extracted names. There are many data sources in PubChem that contribute to this approach, including the European Bioinformatics Institute’s (EBI) SureChEMBL project (REF), the IBM patent collection, and Google Patent extraction.

The query described can be replicated, and the results viewed or exported, using this hyperlink: <https://pubchem.ncbi.nlm.nih.gov/#query=C(CF)(F)F&input_type=smarts&fullsearch=true&page=1>

A similar query, restricting the R groups to non-hydrogens, can be replicated using this hyperlink: <https://pubchem.ncbi.nlm.nih.gov/#query=%5B!H%5DC(C(%5B!H%5D)(%5B!H%5D)F)(F)F&input_type=smarts&fullsearch=true&page=1>

A growth profile of PFAS chemicals over the years 2004-2021 was created by harvesting data from PubChem with the registration time stamp using the REST API. The plot (see Figure S.I.1) shows that the addition of PFAS chemicals into PubChem continues. We might imagine that the growing regulatory concern regarding the toxicity of chemicals in the PFAS class might slow the growth of PFAS chemicals being added to public domain databases (or patents). However, as exemplified by the data flowing into PubChem, there is no evidence that the growth in new PFAS chemicals is slowing at this time.


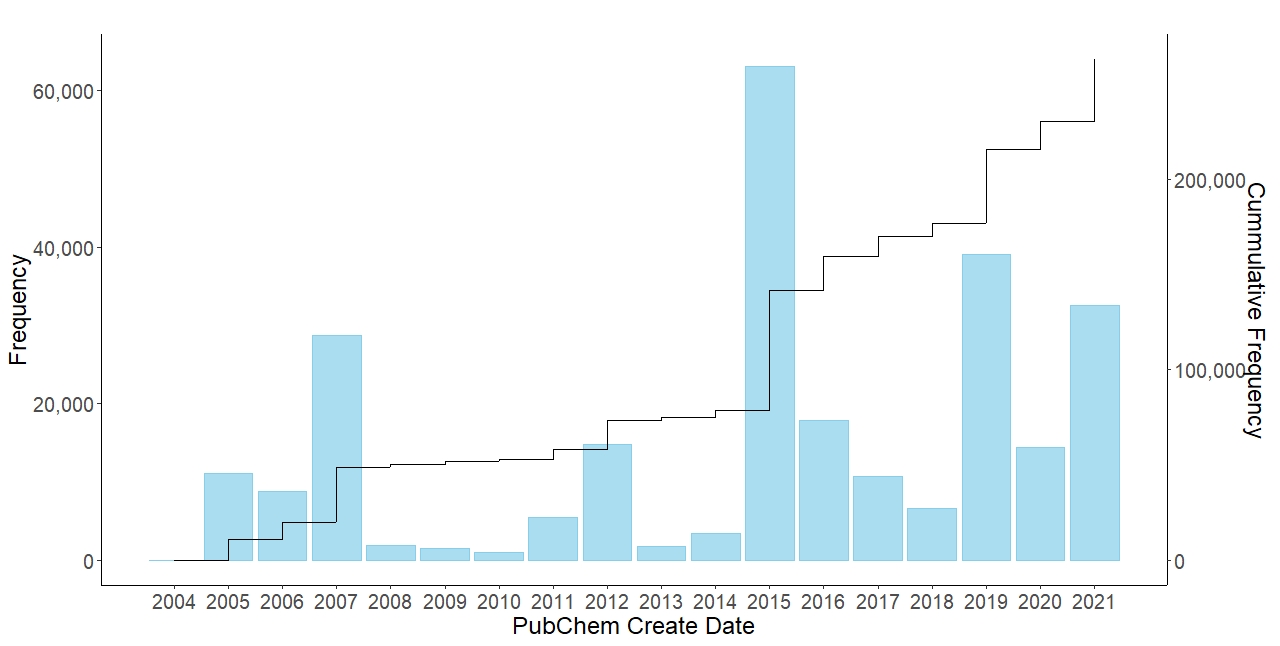


**Figure SI.1**: A plot showing the rate of addition of PFAS chemicals (based on the SMARTS substructural query C(CF)(F)F) as a function of time from 2004-2021.

A similar plot (over the period 2008-2021) is shown in Figure SI.2 for PFAS chemicals in the PFASSTRUCTV4 list. The inclusion of data from public domain sources was initiated in 2014 and continues through our ongoing curation efforts. Our dataset assembly is more controlled in nature than some of the other public domain databases, which influences the rate of growth of our chemical dataset i.e. much slower.
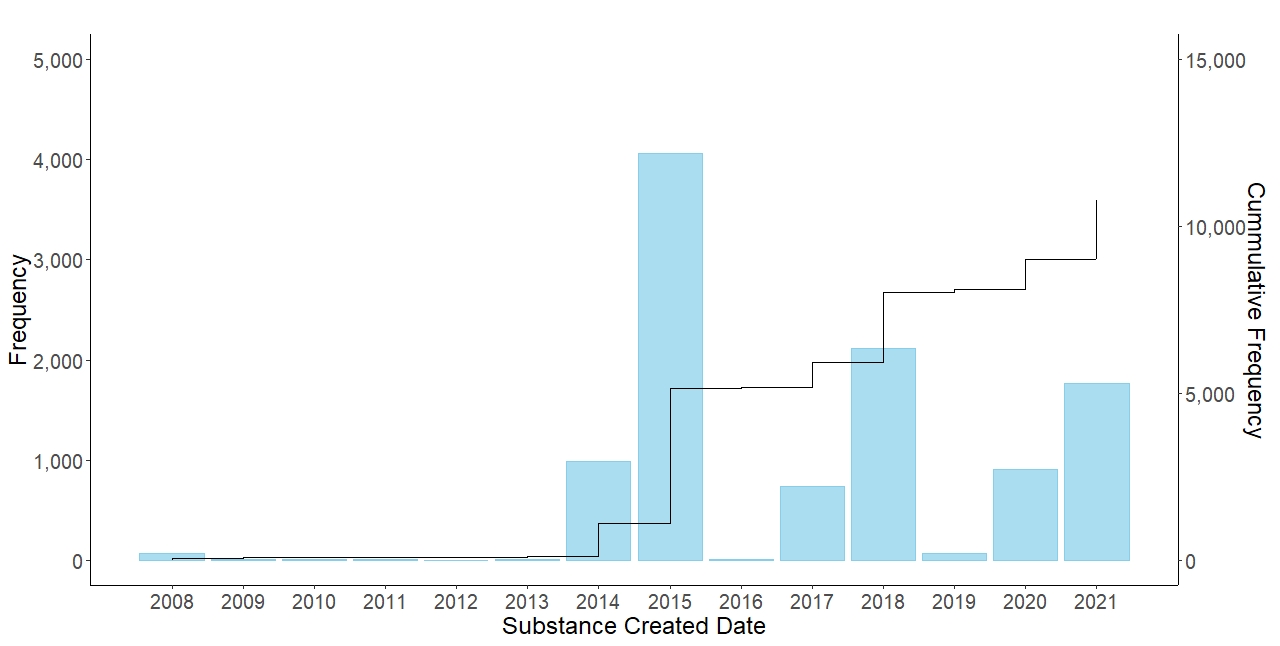


**Figure SI.2**: A plot showing the rate of addition of PFAS chemicals presently listed in the PFASSTRUCTV4 list as a function of time (2008-2021).
